# The COVID-19 pandemic: an underlying factor for increased *Stenotrophomonas maltophilia* infections—A literature review and case study analysis

**DOI:** 10.3389/fmicb.2025.1746742

**Published:** 2026-01-20

**Authors:** Arianna Pompilio, Giovanni Di Bonaventura

**Affiliations:** 1 Laboratory of Clinical and Environmental Microbiology, Department of Medical, Oral and Biotechnological Sciences, “G. d'Annunzio” University of Chieti-Pescara, Chieti, Italy; 2 Laboratory of Clinical Microbiology, Center for Advanced Studies and Technology, “G. d'Annunzio” University of Chieti-Pescara, Chieti, Italy

**Keywords:** co-infections, COVID-19, healthcare-associated infections, intensive care units, *Stenotrophomonas maltophilia*, superinfections

## Abstract

*Stenotrophomonas maltophilia* is increasingly recognized as a major cause of healthcare-associated infections in intensive care units. It presents serious risks for immunocompromised patients and can cause severe lung infections in individuals with cystic fibrosis. Recent studies have documented a rising occurrence of *S. maltophilia* infections among hospitalized COVID-19 patients. However, understanding of these infections in this setting remains limited or inconsistent, with only one review specifically examining *S. maltophilia* infections in COVID-19 patients. This review critically evaluates all relevant studies from the literature, along with a case series, to explore the clinical significance of *S. maltophilia* infections in patients with COVID-19. In particular, the review discusses the prevalence, risk factors, phenotypic traits, clinical consequences, and treatment options for *S. maltophilia* infections in this clinical context.

## 1 Introduction and aim

*Stenotrophomonas maltophilia* is a non-fermenting, Gram-negative bacterium commonly found in the natural environment, where it exists in various reservoirs, such as plants, soil, and animals (Brooke, 2021). Although not inherently virulent, *S. maltophilia* has become an important clinical pathogen over recent decades (Pompilio et al., 2011; Klimkaite et al., 2025), causing disease not only in severely debilitated or immunocompromised patients but also in healthy individuals through community-acquired infections (Brooke, 2021). Reports indicate that infections caused by *S. maltophilia*—including pneumonia, catheter-associated bacteremia/septicemia, osteochondritis, mastoiditis, meningitis, and endocarditis—are associated with high mortality rates ranging from 30% to 70% (Brooke, 2021; Costa et al., 2025). This elevated mortality rate is primarily due to the pathogen's multidrug-resistant profile, which can be acquired in healthcare settings or evolve rapidly within the community (Appaneal et al., 2022; Siew et al., 2025). *S. maltophilia* exhibits inherent resistance to several broad-spectrum antibiotics, such as most β-lactams, fluoroquinolones, aminoglycosides, and trimethoprim.

During the global coronavirus disease 2019 (COVID-19) pandemic, an increase in the incidence of multidrug-resistant (MDR) bacteria has been observed among critically ill patients with COVID-19, mainly due to the overuse of antimicrobial agents. *S. maltophilia* was identified as one of the most common causes of respiratory co-infections, superinfections, and bacteremia in these patients (Ishikawa et al., 2022). The clinical significance of *S. maltophilia* infections is high, primarily due to its intrinsic resistance to commonly used antibiotics and its ability to colonize and form biofilms on medical devices, such as those used for mechanical ventilation (Pompilio et al., 2020; Demirbuga et al., 2024). The isolates from respiratory tract specimens showed the highest rates of multidrug resistance among other bacteria infecting COVID-19 patients (Ishikawa et al., 2022; Langford et al., 2023). Additionally, *S. maltophilia*'s capacity to influence the metabolism and virulence of neighboring microorganisms can play a role in polymicrobial communities, either through antagonistic suppression or symbiotic coexistence. This is evident in the lungs of patients with cystic fibrosis (CF), where it interacts with *Pseudomonas aeruginosa, Staphylococcus aureus*, and *Burkholderia cenocepacia* (Pompilio et al., 2015; Crisan et al., 2024).

The prevalence and risk factors linked to *S. maltophilia* have been rarely studied through case reports during the COVID-19 pandemic. Acquiring this knowledge is crucial for developing evidence-based guidelines to prevent colonization and enhance infection management. This review, therefore, aims to analyze the relevant literature, focusing on recent evidence supporting the role of *S. maltophilia* in COVID-19 patients, and to discuss the limitations of these studies. Accordingly, this narrative review integrates a structured literature search with a critical, qualitative synthesis of the evidence. Although this review includes all reported *S. maltophilia* superinfections in COVID-19 patients, the available evidence is predominantly focused on respiratory infections, particularly pneumonia and ventilator-associated pneumonia (VAP), reflecting the limitations of the existing literature.

## 2 Literature search strategy, selection criteria, and literature included

This review was performed as a narrative review with systematic search elements, aimed at providing a comprehensive and critical overview of the available evidence rather than a formal systematic or scoping review.

The PubMed and MEDLINE electronic databases were searched for relevant articles using a combination of subject terms, free words, and Boolean logical operators: “Stenotrophomonas maltophilia” and “COVID-19”, and (“Stenotrophomonas maltophilia”[MeSH Terms] OR “Stenotrophomonas maltophilia”[Text Word] OR “Stenotrophomonas maltophilia bacteremia”[Supplementary Concept] OR “Pseudomonas maltophilia”[Title/Abstract] OR “Xanthomonas maltophilia”[Title/Abstract] OR “s maltophilia”[Title/Abstract]) AND (“Pneumonia”[MeSH Terms] OR “pneumoni^*^”[Text Word] OR (“pulmonary infect^*^”[Title/Abstract] OR “lung infect^*^”[Title/Abstract]) OR “Respiratory Tract Infections”[MeSH Terms]) AND (“COVID-19”[All Fields] OR “COVID-19”[MeSH Terms] OR “COVID-19 Vaccines”[All Fields] OR “COVID-19 Vaccines”[MeSH Terms] OR “COVID-19 serotherapy”[Supplementary Concept] OR “covid 19 nucleic acid testing”[All Fields] OR “covid 19 nucleic acid testing”[MeSH Terms] OR “covid 19 serological testing”[All Fields] OR “covid 19 serological testing”[MeSH Terms] OR “covid 19 testing”[All Fields] OR “covid 19 testing”[MeSH Terms] OR “sars cov 2”[All Fields] OR “sars cov 2”[MeSH Terms] OR “Severe Acute Respiratory Syndrome Coronavirus 2”[All Fields] OR “NCOV”[All Fields] OR “2019 NCOV”[All Fields] OR ((“coronavirus”[MeSH Terms] OR “coronavirus”[All Fields] OR “COV”[All Fields]) AND 2019/11/01: 3000/12/31[Date – Publication])).

Inclusion criteria were: records with full-text available in English, published (or accepted for publication) between January 1, 2020, and July 15, 2025, with a confirmed diagnosis of COVID-19 by a positive real-time reverse transcription PCR result, and with bacterial infections assessed through culture or molecular analyses of respiratory (i.e., sputum, endotracheal aspirate, and bronchoalveolar lavage) or blood specimens. Consistent with the available literature, most included studies investigated respiratory specimens, while data on non-respiratory infection sites in COVID-19 patients were limited. Studies were excluded if they met any of the following criteria: duplicate reports, conference reports, reviews, or insufficient, missing, or irrelevant data.

The initial search yielded 108 records. The first step removed 47 duplicates. Two authors independently reviewed the 61 titles and abstracts, retrieved full texts for eligibility assessment, and extracted information from these studies. A total of 19 studies were excluded due to insufficient data or failure to align with the review's primary objective (e.g., small patient numbers, inadequate prevalence data, or patients not infected with COVID-19). Additionally, after reviewing the collected articles, six more articles were identified that were not included in the initial screening but met the search criteria.

**Figure 1:**
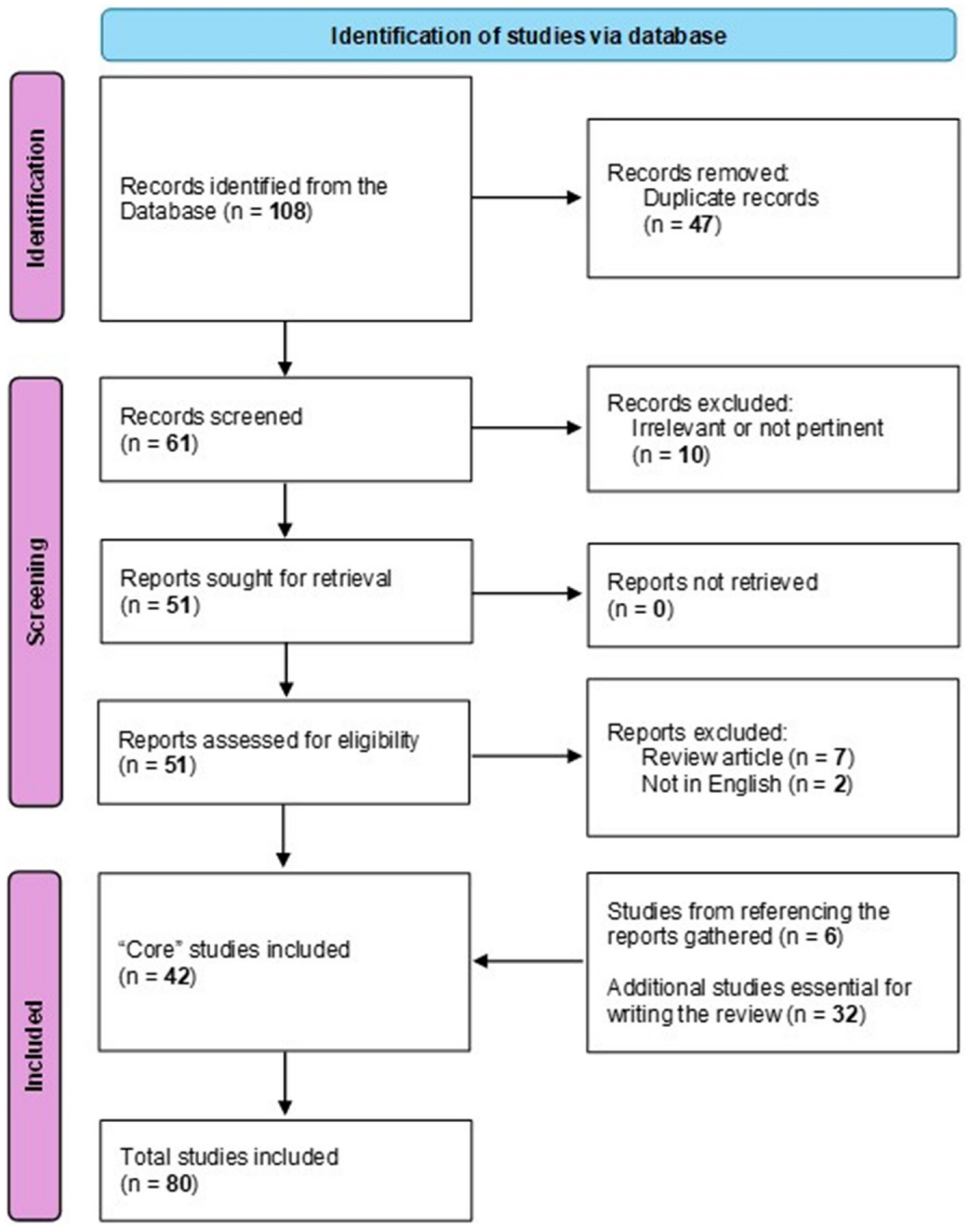
Flow diagram of the literature search and study selection process. Records were identified through PubMed and MEDLINE using predefined search terms and inclusion criteria. Only articles published in English were considered to ensure consistency and reproducibility of data extraction. Studies meeting eligibility criteria were included in the core analysis of *S. maltophilia* infections in COVID-19 patients. In addition, a set of articles that did not meet the screening criteria (e.g., non-prevalence studies, pre-COVID literature, or studies addressing microbiological, mechanistic, or antimicrobial resistance aspects) was included separately as contextual references to support interpretation and discussion. It was not part of the formal screening process.

After applying these criteria, 48 studies comprised the core literature for this review: 31 were prevalence studies (30 retrospective and one prospective), 11 were case reports, and six were phenotypic studies. An additional 32 references that did not meet the predefined screening criteria were included to provide microbiological, mechanistic, and clinical context and were not considered part of the core screened literature (Figure 1).

The 31 prevalence studies were primarily conducted at a single center (23 out of 31, 74.2%), and to a lesser extent, at multiple centers (eight out of 31, 25.8%). A total of 72,100 COVID-19 patients were evaluated for bacterial infections. Twenty-four studies (77.4%) were conducted in Asia, six (19.3%) in Europe, and one (3.2%) in the Americas. Regarding the 11 case reports considered, five (45.4%) were from Asia, four (36.4%) from the Americas, and two (18.2%) from Europe.

## 3 Prevalence of *S. maltophilia* infection in COVID-19 patients

Viral pneumonia can weaken the host's immune system and activate the entry pathways of bacterial pathogens, thereby increasing the risk of bacterial invasion (Hendaus et al., 2015; Hanada et al., 2018). A harmful synergistic effect appears to exist between co-infection with viruses or fungi and hospital-acquired bacterial pneumonia (HABP). Reduced immune responses from a previous infection may render patients more susceptible to developing subsequent HABP, resulting in worse clinical outcomes (Kim et al., 2014).

During the COVID-19 pandemic, *S. maltophilia* has emerged as a notable cause of secondary bacterial infection among Intensive Care Unit (ICU) patients with COVID-19. Evidence from diverse geographic settings consistently shows increased detection of this pathogen, particularly in ventilated and critically ill populations. The most critical studies on this emerging issue are summarized in Table 1.

**Table 1:** Most relevant studies reporting COVID-19-associated *Stenotrophomonas maltophilia* infections.

**Study**	**Study design**	**Country**	**Study period**	**Microbiological diagnosis**	**No. of patients/bacterial strains**	**Hospital wards**	**Main findings**
Li et al., 2020	Retrospective	Wuhan, China	January 2020-March 2020	Culture from respiratory (sputum, endotracheal aspirate, bronchoalveolar lavage fluid), blood, and urine samples	102 COVID-19 patients with SBI. Mean (SD) age: 66.2 (11.2) years. Males: 66.7%.	NS	The most commonly isolated bacteria were *A. baumannii* (35.8%), *K. pneumoniae* (30.8%), and *S. maltophilia* (**6.3%**)
Sang et al., 2021	Retrospective, multicenter	Two hospitals in Wuhan and one in Guangzhou, China	January 2020-April 2020	Culture from the lower respiratory tract, blood, and other body fluid specimens	190 COVID-19 patients. Mean age: 62.7 years. Males: 67.4%.	ICU	*S. maltophilia* overall prevalence was **9.9%**, secondary only to *K*. *pneumoniae* (24.5%) and *A*. *baumannii* (21.8%). It was the most commonly isolated (22.5%) at the Guangzhou hospital. *S. maltophilia* was primarily from respiratory samples (95.4%), followed by blood (1.8%), and other specimens (2.8%).
Yang et al., 2021	Retrospective	Beijing, China	January 2020-April 2020	Culture and real-time PCR from sputum and bronchoalveolar lavage	20 COVID-19 patients: - 7 in the severe group (median age: 39 years; IQR: 36–67 years) - 13 in the critical group (median age: 69 years; IQR: 64–80 years)	ICU	Most common pathogens at culture: - *B. cepacia* (18.8%) and *S. maltophilia* (**15.6%**) in the critical group - No growth in the severe group Most common pathogens at real-time PCR: - *S. maltophilia* (**45.5%**) and *S. aureus* (42.4%) in the critical group - *S. maltophilia* and *P. aeruginosa* (**28%** each) in the severe group
Zhang et al., 2023	Retrospective	China	October 2022-January 2023	Culture from the sputum	163 (84 COVID-19, and 79 non-COVID-19) elderly LRTI consecutive patients. Median (IQR) age: 73.5 (67.0–79.0) years. Males: 57.9%.	ICU	Higher frequency of *S. maltophilia* in the COVID-19 group than in the non-COVID-19 group (**12.2%** ***vs***. **3.1%**)
Nakagawara et al., 2023	Retrospective, multicenter	Japan COVID-19 Task Force (composed of more than 70 institutions nationwide in Japan)	April 2020-May 2021	NS	1,863 COVID-19 patients		12.2% patients had concomitant bacterial infections. *S. aureus* and *K. pneumoniae* were the most frequently isolated microorganisms in respiratory bacterial co-infections. *S. maltophilia* is among the most frequently isolated (**11.1%**) microorganisms in secondary infections in VAP patients, along with *P. aeruginosa* (20.6%) and *S. aureus* (15.9%).
Morimoto et al., 2024	Retrospective	Japan	April 2020-September 2021	Culture of sputum, lower respiratory tract aspiration, and blood culture showing the same bacteria as in the sputum culture	68 COVID-19 pneumonia patients: 23 VAP, and 45 non-VAP. Median age: 58 years (IQR, 51.75–66). Males: 83.8%.		*S. maltophilia* and *P. aeruginosa* were the most prevalent (**30.4%** and 26.1%, respectively)
Son et al., 2021	Retrospective, multicenter	Republic of Korea (10 hospitals)	February 2020-May 2020	Culture from sputum, blood, urine, and stool specimens	152 patients with COVID-19. Mean age (IQR): 68 years (62–77). Males: 55%.	Long-term care facility, outpatient clinic visit, dialysis clinic.	47 patients had positive cultures. MDROs were detected in 28% of patients with culture data and 8.6% of the entire cohort. *S. maltophilia* was the most common MDRO (**38.4%**).
Choi et al., 2023	Retrospective, propensity-score-matched, multicenter	Two university hospitals, Seoul, South Korea	2011-2021	Culture from respiratory specimens (sputum, bronchoalveolar lavage, endotracheal secretions, and pleural fluid)	17,250 patients with HAP. Mean (SD) age: 65.5 (15.4) years. Males: 65.4%.		Preceding SARS-CoV-2 infection was not associated with *S. maltophilia*. The incidence of HABP caused by *S. maltophilia* was associated with a high APACHE II score and high 28-day all-cause mortality (aOR 1.32, 95% CI 1.05–1.66), in contrast to HABP caused by *P. aeruginosa*.
Huang et al., 2022	Retrospective	Taipei, Taiwan	January 2020-July 2021	NS	204 COVID-19 patients		A total of 38 bacterial infection episodes occurred in 23 inpatients. The most frequently isolated bacteria were *Acinetobacter* spp. (50%), followed by *S. maltophilia* (**36.8%**). Of 14 patients with *S. maltophilia* infections, three were admitted to the ICU, and 11 had previously received carbapenem therapy.
Signorini et al., 2021	Retrospective	Brescia, Italy	February 2020-May 2020	Culture from bronchoalveolar lavage and blood samples	92 patients with COVID-19. Median age (IQR): 62.0 years (56.3–67.8). Males: 87%.	ICU	The most common pathogens responsible for VAP were *P. aeruginosa* (34.7%) and *S. maltophilia* (**18.7%**).
Romanelli et al., 2023	Retrospective	Bari, Italy	January 2018-December 2019 (pre-COVID-19), and March 2020-December 2021 (COVID-19)	Culture from blood, urine, and tracheobronchial aspirate	1,905 patients (619 COVID-19, and 1,286 non-COVID-19). Median (IQR) age: - COVID-19 patients: 66 (58–74) for females, and 66 (56–73) for males - non-COVID-19 patients: 65 (48–74) for females, and 64 (49–75) for males Female/male: 0.613.	COVID-19 and non-COVID-19 ICUs	Increased *S. maltophilia* prevalence during the pandemic compared to before the pandemic, in respiratory samples (**7.98%** ***vs***. **4.53%**, respectively; *p < * 0.05). Increased, although not at significant levels, were observed in urine (0.17% *vs*. 0.08%, respectively; p>0.05) and blood (1.08% *vs*. 0.37%, respectively; p>0.05) samples.
Foschi et al., 2021	Retrospective, multicenter	Bologna, Italy	10 March 2020-30 December 2020	Culture and multiplex PCR on respiratory samples (i.e., bronchial aspirates and bronchoalveolar lavages)	178 critically ill COVID-19 patients	ICUs	Culture was positive in 34.3% of samples; *S. maltophilia* was isolated in **3.4%** of cases. Multiplex PCR was positive in **40%** of samples; it missed some pathogens not included in the molecular panel but detected by culture, especially *S. maltophilia*.
Burastero et al., 2022	Retrospective	Modena, Italy	February 2020-March 2022	NS	23 critically ill COVID-19 patients with difficult-to-treat VAP infections caused by non-fermenter GNB. Median (IQR) age: 69 years (64–76.5). Males: 70%.	ICUs	Isolation rate: 82.6% for *Pseudomonas* spp., **26%** for *S. maltophilia*, and 8.6% for *B. cepacia*. 50% of *S. maltophilia* isolates were resistant to trimethoprim/sulfamethoxazole. The 30-day mortality rates were 61.1% for VAPs caused by *Pseudomonas* spp., 50.0% for *B. cepacia*, and 16.0% for *S. maltophilia*.
Rouzé et al., 2021	Retrospective, multicenter	36 European centers (28 in France, 3 in Spain, 3 in Greece, 1 in Portugal, and 1 in Ireland)	March 2016-May 2020	Culture from respiratory samples (endotracheal aspirate, bronchoalveolar lavage)	1,576 patients: - group 1: 568 with SARS-CoV-2 - group 2: 482 with influenza - group 3: 526 without viral infection Mean age (IQR): 64 (55–71) for group 1; 62 (53–71) for group 2; 65 (55–74) for group 3. Males were: 71.7% (group 1), 61.8% (group 2), 67.4% (group 3).	ICUs	GNB were responsible for most (82% to 89.7%) of VA-LRTI. There was no difference in the prevalence of microorganisms among the groups tested. *S. maltophilia* was comparably isolated in **3.5%** COVID-19 patients, **2.1%** of influenza pneumonia patients, and **5.3%** of patients with no viral infections.
Blonz et al., 2021	Retrospective, multicenter	France (7 general hospitals)	March 2020-May 2020	Culture from respiratory samples (i.e., bronchoalveolar lavage, endotracheal aspirate, or pleural fluid)	194 patients with invasive mechanical ventilation for COVID-19. Mean (SD) age: 63.9 (11.4) years. Males: 78.2%.	ICUs	*S. maltophilia* was isolated, only after 5 days of invasive mechanical ventilation, from **3.9%** of patients. Half of *S. maltophilia* infections were polymicrobial.
Baiou et al., 2021	Retrospective	Doha, Qatar	March 2020-June 2020	Culture from respiratory, blood, and urine samples	234 COVID-19 patients with (*n =* 74) or without (*n =* 138) at least one MDR GNB isolated during their ICU admission. Mean age (IQR): 49 years (40–60). Males: 90.6%.	ICU	*S. maltophilia* was the most frequently isolated GNB (**24.5%**), followed by *K. pneumoniae* (23.5%), and *E. cloacae* (18.4%)
Garcell et al., 2023	Retrospective	Qatar	2012-2019 (pre-COVID-19), and 2020-2021 (during the COVID-19 pandemic)	Culture from the respiratory tract (sputum, tracheal aspirate, and other respiratory samples)	155 patients with HAI, of whom 130 (85.5%) were identified during the COVID-19 period	Medical-surgical ICU	Higher frequency of *S. maltophilia* during the COVID-19 pandemic period, although not at a significant level (**6.8%** ***vs***. **4.3%**)
Senok et al., 2021	Retrospective, multicenter	All Dubai Health Authority hospitals and one in Umm Al Quwain, United Arab Emirates	February 2020-July 2020	Culture from respiratory samples (sputum, endotracheal aspirate, bronchoalveolar lavage), blood, urine, wound swab, and others	29,802 COVID-19 patients. Mean (SD) age: 49.3 (12.5) years. Males: 84.2%.	NS	A total of 392 (1.3%) patients had co-infections. *S. maltophilia* was more prevalent in subsequent cultures than in the first cultures (**35.7%** ***vs***. **4.8%**, respectively). *S. maltophilia* was never isolated in patients positive for more than one organism.
Hafiz et al., 2022	Retrospective	Riyadh, Saudi Arabia	January 2019-December 2021	Culture from blood (central and peripheral lines), respiratory samples (sputum and endotracheal aspirate), urine (mid-stream urine, indwelling and in/out catheters), and miscellaneous (abscess, wound, tissue, body fluid, and device) sources	393 *S. maltophilia* isolates: 209 from ICU patients, and 184 from non-ICU patients. Males: 58.8%.	ICU (53.2%), Emergency (8.7%), Outpatient Clinic (5.8%), Wards (32.3%).	Nearly half (50.7%) of the ICU patients were coinfected with GNB (64%; e.g., *P. aeruginosa, K. pneumoniae, K. oxytoca, A. baumannii, E. cloacae, E. coli)* and SARS-CoV-2 (48%). In non-ICU patients, the prevalence of *S. maltophilia* increased during the pandemic in both respiratory (**38.7%** in 2019 *vs*. **47.5%** in 2021; *p < * 0.05) and blood samples (17.7% in 2019 *vs*. 31.2% in 2021; *p* = 0.01). The same trend was observed among ICU patients for blood samples only (**7.4%** in 2019 *vs*. **13.7%** in 2021; *p* = 0.01).
AlFonaisan et al., 2024	Retrospective	Saudi Arabia	2004-2022	Culture analysis from diverse sources: blood, respiratory samples, urine (including indwelling catheters), abscesses, wounds, tissues, and body fluids	4,446 *S. maltophilia*-positive, nonduplicated patients	Adult and surgical ICU (32.2%), family medicine clinics, eye clinic, orthopedics clinics, immunology diseases clinic, nephrology clinic, colorectal clinic, and otolaryngology clinic (29.8%), cardiology ward (15.9%), and pediatric ICU (10.8%)	Overall, the number of *S. maltophilia* infections increased by approximately 50% from 2018 to 2022. The same trend was observed for ICU and non-ICU patients.
Raad et al., 2023	Retrospective	Beirut, Lebanon	March 2020-March 2021	Culture from sputum and tracheal aspirates when on mechanical ventilation	123 COVID-19 patients. Mean (SD) age: 66.5 (13.5) years. Males: 74%.	2 separate ICUs	The most common isolated pathogen was *S. maltophilia* (**16.2%**), followed by *Enterobacterales* spp. (9.5%), and *Pseudomonas* spp. (6%). Higher median death rate in *S. maltophilia-*infected patients (60% *vs*. 40%). *S. maltophilia* superinfection increased the chances of long-term complication rate at discharge (*vs*. not infected patients): - tracheostomy (28.6% *vs*. 4.8%) - oxygen dependence (57% *vs*. 42%)
Shmoury et al., 2023	Retrospective	Lebanon	March 2020-September 2021	Culture of sputum or deep tracheal	1,674 patients admitted with COVID-19. 9.5% developed one or more bacterial respiratory infections with an identifiable causative organism within 42 days of hospitalization. Males: 69%. Median (IQR) age: 68 (16) years.		*S. maltophilia* and *K. pneumoniae* were the most encountered pathogens, each accounting for **14%** of all infections. *S. maltophilia* (**19%**) was the most common pathogen among subjects with HAP. *S. maltophilia* was isolated within hospital days 3-28, with the highest isolation during days 15-28. *S. maltophilia* was the most common among VAP cases (**24%**), where it was significantly more encountered than in non-VAP patients. The incidence of *S. maltophilia* in COVID-19 VAP was higher compared to pre-COVID-19 (**24%** ***vs***. **6%**; *p =* 0.001).
Shiralizadeh et al., 2025	Retrospective	Hamadan, Iran	September 2020- August 2021	Culture from blood, sputum, tracheal aspiration, and urine	207 COVID-19 patients	ICU	*K. pneumoniae* and *A. baumannii* were the most prevalent (33.3% and 28.1%, respectively). *S. maltophilia* prevalence was **1.45%**.
Rahaman et al., 2022	Prospective	Bangladesh	NA	Metagenomic analysis of nasopharyngeal swab samples	17 COVID-19 patients (10 vaccinated, and 7 non-vaccinated), and 2 COVID-19-negative patients	NS	*S. maltophilia* co-infection was found in 10 out of 17 (**58.8%**) COVID-19 patients. *S. maltophilia* was found in both vaccinated and non-vaccinated COVID-19 patients (**9.38% and 5.5%**, respectively). *S. maltophilia* relative abundance ranged from 0.9% to 22.1%.
Ismaeil et al., 2023	Retrospective	Malaysa	April-October 2019 (pre-pandemic) and April-October 2020 (during pandemic)		578 patients: 321 in pre-COVID-19, and 257 during COVID-19	Medical, surgical, and critical/ICU wards	*S. maltophilia* was identified exclusively during the COVID-19 period, with a prevalence rate of **6.9%**
Cornejo-Juárez et al., 2023	Retrospective	Mexico City, Mexico	Pre-pandemic (2018, 2019, and the first 3 months of 2020) and pandemic period (April–December 2020 and all of 2021) period	NS	639 patients with HAIs: 381 during the pre-pandemic period and 258 during the pandemic period. Mean (SD) age: 49.9 (17.8) years; patients were older in the pandemic period *vs*. the pre-pandemic period: 51.7 (17.5) *vs*. 48.8 (18). Males: 52.6%.	COVID-19 ICU	*S. maltophilia* was more frequent in the pandemic period *vs*. the pre-pandemic period (**10.9%** ***vs***. **4.5%**; *p =* 0.001).
Önal et al., 2023	Retrospective	Turkey	January 2018-December 2021	Culture from blood, central venous catheters, respiratory specimens, urine, and urinary catheters	8,157 adult patients (≥18 years) diagnosed with HAIs. Patients were divided into two groups: those from the pre-pandemic period (2018-2019) and those from the pandemic period (2020-2021).	ICUs (anaesthesiology and reanimation, general surgery, cardiovascular surgery, neurosurgery, and the ICU of COVID-19 patients)	*S. maltophilia* bacteraemia episode rates in the ICU of COVID-19 patients were significantly higher than in the other ICUs, both during (**3.038** ***vs***. **1.297**; *p < * 0.01) and before (**1.297** ***vs***. **0.205**; *p < * 0.0001) the COVID-19 pandemic

*S. maltophilia* prevalence values are bolded to make comparison across studies easier.

SBI, secondary bacterial infection; SD, standard deviation; NS, not specified; RT-PCR, real-time PCR; IQR, interquartile range; ICU, intensive care unit; LRTI, lower respiratory tract infections; VAP, ventilator-associated pneumonia; MDROs, multidrug-resistant organisms; HAP, hospital-acquired pneumonia; HABP, hospital-acquired bacterial pneumonia; APACHE, acute physiology and chronic health evaluation; aOR, adjusted-odds ratio; GNB, Gram-negative bacteria; VA-LRTI, ventilator-associated lower respiratory tract infection; HAI, hospital-acquired infection; MDR, multidrug-resistant; NA, not applicable.

### 3.1 East Asia

Early evidence emerged from China, where Li et al. (2020) conducted an extensive retrospective cohort study of 1,495 hospitalized COVID-19 patients in Wuhan. Secondary bacterial infections occurred in 6.8% of cases and were associated with a high mortality rate. Gram-negative organisms predominated, with *S. maltophilia* accounting for 6.3% of isolates, ranking after *Acinetobacter baumannii* and *Klebsiella pneumoniae*. A subsequent multicenter retrospective ICU study across three Chinese hospitals confirmed a high burden of secondary infections (86.6%), identifying *S. maltophilia* as the third most frequent pathogen overall and the leading isolate at one center (Sang et al., 2021). Yang et al. (2021), in a retrospective analysis of patients at Beijing Hospital, reported that *S. maltophilia* was the most common co-infecting organism in both severe and critical COVID-19 cases. This study also demonstrated that multiplex real-time PCR significantly increased detection rates compared with culture-based methods, underscoring the influence of diagnostic approach on reported prevalence. Zhang et al. (2023) further demonstrated a higher prevalence of *S. maltophilia* also among early COVID-19 patients with lower respiratory tract infections than among non-COVID-19 controls.

Several studies from Japan further corroborated these findings. Nakagawara et al. (2023) identified *S. maltophilia* as one of the three most frequent causes of hospital-acquired secondary infections, which were strongly associated with disease severity and mortality. Similarly, Morimoto et al. (2024) reported *S. maltophilia* as the leading cause of VAP in patients with severe COVID-19.

In South Korea, multicenter retrospective data demonstrated that *S. maltophilia* was the most common multidrug-resistant organism (MDRO) among COVID-19 patients with co-infections, often isolated late in the disease course (Son et al., 2021). Longitudinal surveillance data further linked *S. maltophilia*-associated HABP to high illness severity and 28-day mortality, as assessed by APACHE II (Acute Physiology and Chronic Health Evaluation II) score, despite a lower incidence compared with *P. aeruginosa* (Choi et al., 2023).

In Taiwan, Huang et al. (2022) conducted a retrospective hospital-based study and identified *S. maltophilia* as the second most common bacterial co-pathogen in COVID-19 patients with hospital-acquired infections (HAIs).

### 3.2 Europe

Several observational and retrospective studies from Italy highlighted the prominence of *S. maltophilia* in ICU settings. Signorini et al. (2021) described a high incidence of VAP and bloodstream infections during the first pandemic wave, with *S. maltophilia* accounting for nearly one-fifth of VAP cases. Romanelli et al. (2023) further demonstrated that SARS-CoV-2 infection was specifically associated with increased respiratory isolation of *S. maltophilia*, but not with urinary or bloodstream infections. Foschi et al. (2021) further validated molecular diagnostics for *S. maltophilia* detection, demonstrating high sensitivity and reduced turnaround time. Burastero et al. (2022) conducted a single-center retrospective cohort study evaluating treatment outcomes for VAP caused by non-fermenting Gram-negative bacteria. *S. maltophilia* was frequently involved in polymicrobial infections, exhibited high resistance to trimethoprim/sulfamethoxazole (TMP/SMX), yet was associated with lower mortality compared with other non-fermenters.

In France, Rouzé et al. (2021) conducted a multicenter, prospective, observational study comparing ventilator-associated lower respiratory tract infections among patients with COVID-19, influenza, and non-viral pneumonia. The prevalence of *S. maltophilia* was comparable across groups, suggesting that its occurrence may be more closely associated with mechanical ventilation than with SARS-CoV-2 itself. Similarly, Blonz et al. (2021) reported low but consistent isolation rates, predominantly in late-onset VAP.

### 3.3 Middle East

In Qatar, Baiou et al. (2021) and Garcell et al. (2023) identified *S. maltophilia* as the most frequent Gram-negative isolate among critically ill COVID-19 patients, surpassing *Klebsiella pneumoniae* and *Enterobacter cloacae*. Similarly, in the United Arab Emirates, a multicenter retrospective study revealed a marked increase in *S. maltophilia* isolation in later cultures during hospitalization, highlighting its role as a secondary or late-emerging pathogen (Senok et al., 2021).

Several studies from Saudi Arabia have demonstrated temporal shifts in the epidemiology of *S. maltophilia*. Hafiz et al. (2022) reported stable ICU infection rates but a significant increase among non-ICU patients during the pandemic, particularly in respiratory and bloodstream infections. AlFonaisan et al. (2024), however, observed a significant rise in ICU-associated *S. maltophilia* infections during the same period.

In Lebanon, two retrospective studies provided focused analyses. Raad et al. (2023) identified *S. maltophilia* as the leading cause of nosocomial pneumonia in critically ill COVID-19 patients, with high recurrence and mortality rates. Shmoury et al. (2023) confirmed its predominance in HABP and VAP, with a significantly higher incidence compared to the pre-pandemic period.

In Iran, Shiralizadeh et al. (2025) reported a comparatively low prevalence (1.3%) of *S. maltophilia* in ICU patients, illustrating regional variability.

### 3.4 South and Southeast Asia

An observational study from Pakistan reported isolation of *S. maltophilia* from COVID-19 patients with invasive aspergillosis, raising questions about its pathogenic role versus colonization in polymicrobial contexts (Nasir et al., 2020).

In Bangladesh, Rahaman et al. (2022) employed genomic and metagenomic approaches, demonstrating frequent polymicrobial co-infections involving *S. maltophilia*. This study uniquely highlighted its consistent co-detection with other nosocomial pathogens and reinforced the polymicrobial nature of COVID-19-associated infections (Jacob et al., 2022).

Studies from Thailand (Satjawattanavimol et al., 2023) and Malaysia (Ismaeil et al., 2023) identified *S. maltophilia* as a contributor to early bacterial co-infections and hospital-acquired infections, respectively, both of which are associated with increased mortality risk.

### 3.5 Americas

In Mexico, Cornejo-Juárez et al. (2023) conducted a retrospective analysis in an oncology hospital. They documented a significant increase in *S. maltophilia* isolation during the pandemic, particularly among immunocompromised cancer patients. Notably, uncommon non-fermenters were identified exclusively in COVID-19 patients, suggesting shifts in ICU microbial ecology.

**Figure 2:**
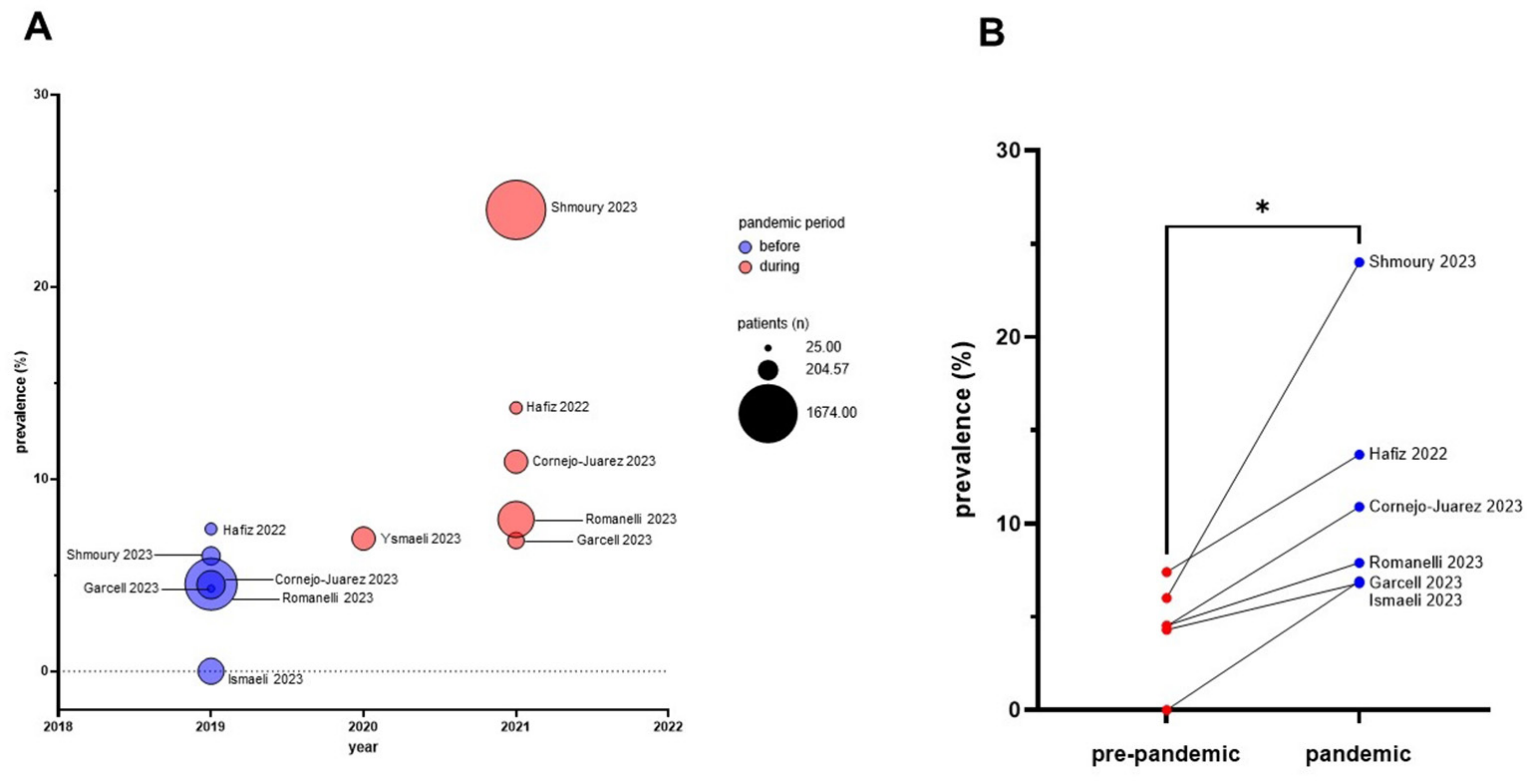
Prevalence of *S. maltophilia* infections observed before and during the pandemic across published studies. **(A)** The x-axis indicates the year the study was conducted, while the y-axis shows the prevalence of *S. maltophilia* infections. The bubble size corresponds to the number of COVID-19 patients enrolled in each study, and the color scale distinguishes between the pre-pandemic period (violet; 2019) and the pandemic period (red; 2020–2022). **(B)** Comparative analysis of *S. maltophilia* prevalence before and during the pandemic, as assessed in six studies. *p < 0.05 at unpaired t-test, mean prevalence: 4.4% *vs*. 11.7%, for “pre-pandemic” and “pandemic” periods, respectively.

**Figure 3:**
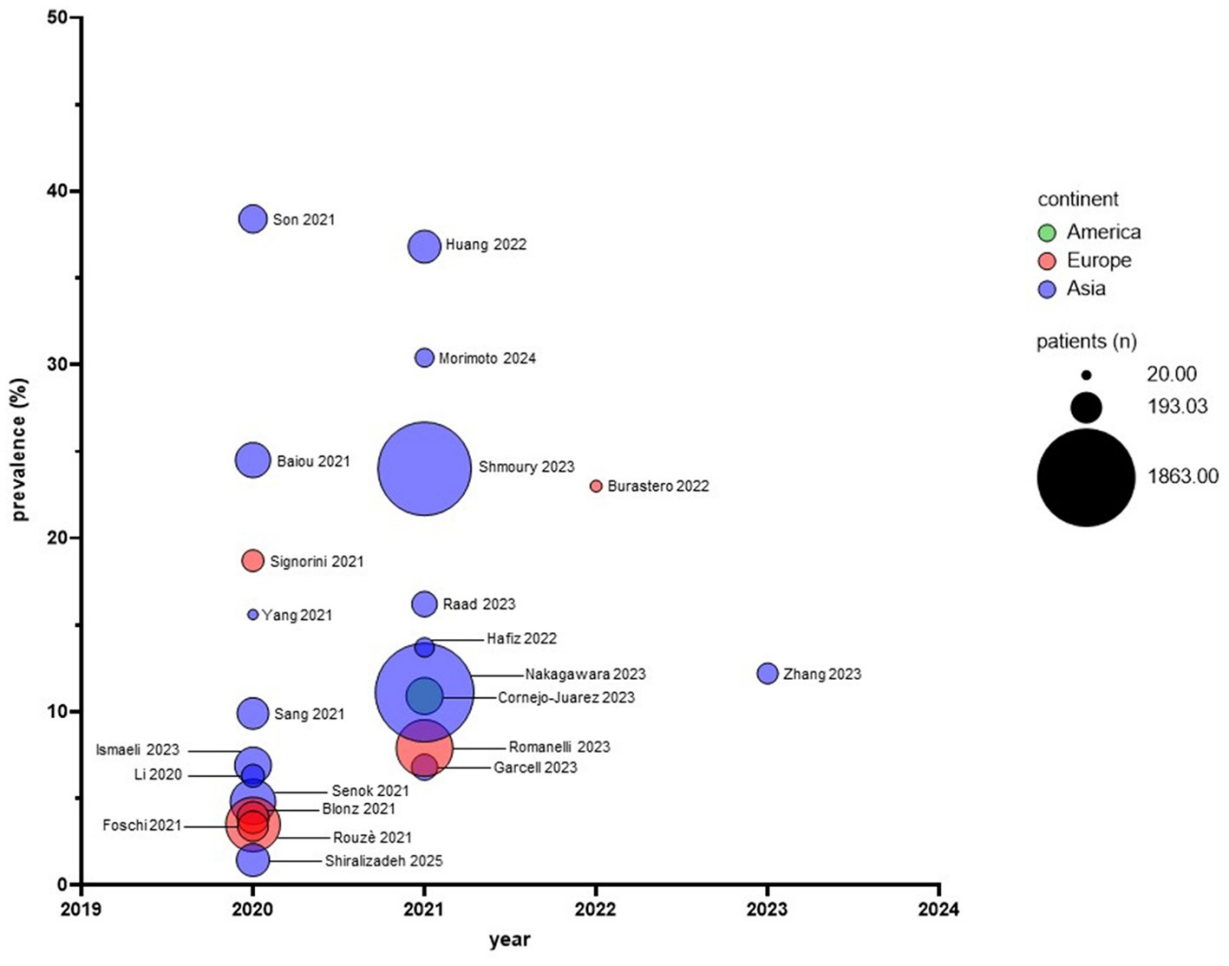
Prevalence of *S. maltophilia* infections observed during the pandemic across 24 studies. The x-axis shows the year each study was conducted, while the y-axis displays the prevalence of *S. maltophilia* infections. The bubble size indicates the number of COVID-19 patients enrolled in each study, and the color scale distinguishes the continents where the studies were conducted.

Across regions and study designs, several consistent trends emerge from the reviewed studies:

- The prevalence of *S. maltophilia* increases during the pandemic period (Burastero et al., 2022; Hafiz et al., 2022; Garcell et al., 2023; Romanelli et al., 2023; Shmoury et al., 2023; Cornejo-Juárez et al., 2023). Considering all the studies, the average prevalence significantly rose from 4.4% to 11.7% (during pre-pandemic and pandemic periods, respectively; *p* = 0.03, unpaired *t*-test) (Figure 2).
- The prevalence of *S. maltophilia* among COVID-19 patients ranges from 1.4% (Shiralizadeh et al., 2025) to 38.4% (Son et al., 2021) (Figure 3). Only one study (Musuroi et al., 2024) reported no isolation of *S. maltophilia*, which may be due to contact measures implemented during the pandemic or to criticisms of the study's methodology (see the “Criticisms and limitations of the reviewed studies” section).
- In COVID-19 patients, *S. maltophilia* can be co-isolated with other bacterial pathogens (Blonz et al., 2021; Burastero et al., 2022; Rahaman et al., 2022; Jacob et al., 2022), such as epidemiologically significant extended-spectrum β-lactamase (ESBL)-producing *K. pneumoniae*, methicillin-resistant *S. aureus* (MRSA), MDR *A. baumannii* (Nasir et al., 2020), and carbapenem-resistant *P. aeruginosa* (Son et al., 2021), highlighting the need to assess the true clinical significance of *S. maltophilia* in these patients.
- Infections caused by *S. maltophilia* are associated with disease severity and high mortality rates (Burastero et al., 2022; Nakagawara et al., 2023; Choi et al., 2023; Raad et al., 2023; Satjawattanavimol et al., 2023), despite variable prevalence across settings, emphasizing the need to evaluate *S. maltophilia* co-infections and to provide timely, appropriate antimicrobial treatment for COVID-19 patients. However, more research is needed to clarify the directly attributable mortality risk factors for *S. maltophilia* pneumonia and the disease consequences of COVID-19 infection.
- *S. maltophilia* is more prevalent in patients with VAP than in those without VAP (Shmoury et al., 2023), indicating that mechanical ventilation is a risk factor for infection.
- Molecular diagnostic techniques substantially increase *S. maltophilia* detection rates compared with culture-based methods (Yang et al., 2021; Rahaman et al., 2022).

## 4 Case reports

Various cases of *S. maltophilia* infection have been reported as complications in COVID-19 pneumonia within the medical literature. Here, all case reports published to date were grouped primarily by clinical setting, and the findings are summarized in Table 2.

**Table 2:** Case reports of COVID-19-associated *Stenotrophomonas maltophilia* infections.

**Case report**	**Study period**	**Country**	**Patient's gender and age**	**Microbiological diagnosis**	**Underlying diseases**	**Antibiotic therapy (before and after *S. maltophilia* diagnosis)**	**Hospital ward**	**Outcome**
Ishikawa et al., (2022)	2021	Japan	Female 52 years	*S. maltophilia* VAP and bacteremia (day 8 from sputum and blood; with CAPA)	Systemic lupus erythematosus, rheumatoid arthritis, liver cirrhosis, and interstitial pneumonia	Before: ceftriaxone, meropenem After: TMP/SMX, voriconazole	ICU (day 8, intubated)	Improved
Ishikawa et al., (2022)	2021	Japan	Male 79 years	*S. maltophilia* VAP (day 9 from sputum, with *K. aerogenes*, and *E. faecalis*)	Dementia	Before: ceftriaxone After: levofloxacin, TMP/SMX, and meropenem	ICU (day 6, intubated)	Improved (day 56)
Ishikawa et al., 2022	2021	Japan	Male 80 years	*S. maltophilia* VAP (day 8 from sputum, considered colonizer; with CAPA and *C. glabrata*)	HTN, acute myocardial infarction	Before: piperacillin/tazobactam After: meropenem, TMP/SMX, levofloxacin, and minocycline (for TMP/SMX-induced acute kidney injury)	ICU (day 2, intubated)	Death (day 28, due to invasive aspergillosis)
Ishikawa et al., 2022	2021	Japan	Male 48 years	*S. maltophilia* VAP (day 10 form sputum; monomicrobial)	HTN, DM, and obesity	Before: piperacillin/tazobactam After: TMP-SMX	ICU (day 1, intubated)	Improved (day 30)
Su et al., 2020	2020	USA	Male 72 years	*S. maltophilia* VAP (day 10 from sputum; monomicrobial)	Guillain-Barré syndrome, HTN, CAD, alcohol abuse	Before: none After: TMP/SMX	ICU (day 3, intubated)	ICU stay with severe weakness
Darvishnia et al., 2021	2020	Iran	Male 71 years	*S. maltophilia* VAP (day 8 from sputum; monomicrobial)	Hodgkin's lymphoma, HTN,	Before: meropenem, vancomycin, linezolid, and imipenem After: colistin, TMP/SMX	ICU (day 1, intubated)	Partial recovery (day 31)
Pek et al., 2021	2020	Mexico	Male 60 years	*S. maltophilia* VAP and bacteremia (day 14 from tracheal aspirate, and day 21 from blood; monomicrobial)	Asthma, HLD, and elevated BMI	Before: Piperacillin/tazobactam, vancomycin, cefepime, meropenem After: TMP/SMX; shortly developed hyperkalemia and volume overload refractory to intravenous furosemide; septic shock on day 7 of therapy	ICU (day 10, intubated)	Death (day 43)
Kuwahara et al., 2022	2021	Japan	Male 50 years	*S. maltophilia* from sputum (day 4, considered colonizer), and bacteremia (day 25 from blood collected at a CVC; monomicrobial)	No relevant medical history	Before: ceftriaxone, cefozopran, meropenem After: TMP/SMX	ICU (day 2, intubated)	Cured (day 53)
Mohamed et al., 2020	2020	USA	Male 64 years	*S. maltophilia* VAP (day 5 from sputum; monomicrobial)	DM, HTN, ESRD, post-renal transplant recipient	Before: cefepime After: TMP/SMX stopped for acute kidney injury (day 7)	ICU (day 3, intubated)	Death (day 12)
Öz et al., 2020	2020	Turkey	Male 61 years	*S. maltophilia* VAP (day 7 from sputum, with *P. aeruginosa*)	COPD, bronchiectasis, previous TB, and bilateral upper lobectomies. Tracheostomy and mechanical ventilation for 2 years.	Before: piperacillin/tazobactam, meropenem After: colistin and fosfomycin	ICU (intubated)	Cured (day 27)
Radici et al., 2023	2021	Italy	Male 44 years	*S. maltophilia* (day 21 from sputum with CAPA; day 30 from bronchoalveolar lavage with *K. pneumoniae*)	Refractory mantle cell lymphoma	Before: piperacillin/tazobactam After: moxifloxacin, amphotericin B (replaced by isavuconazole)	ICU (day 9)	Cured
Bilgic et al., 2021	2020	NS	Male 42 years	*S. maltophilia* endophthalmitis (day 5 from vitreous fluid; monomicrobial)	No systemic comorbidities. Dental caries, previous COVID-19 pneumonia.	Before: prophylactic broad-spectrum antibiotics for dental extraction After: cefepime		Cured (3 months after vitrectomy)
Alharami et al., 2024		USA	Female 15 years	*S. maltophilia* from oral ulcers; monomicrobial	None	Before: cephalexin After: TMP/SMX		Cured

VAP, ventilator-associated pneumonia; CAPA, COVID-19-associated pulmonary aspergillosis; TMP/SMX, trimethoprim/sulfamethoxazole; ICU, intensive care unit; HTN, hypertension; DM, diabetes; CAD, coronary artery disease; HLD, hyperlipidaemia; BMI, body mass index; CVC, central venous catheter; ESRD, end-stage renal disease; COPD, chronic obstructive pulmonary disease; TB, tuberculosis; NS, not stated.

### 4.1 ICU and severe respiratory COVID-19 settings

Most reported *S. maltophilia* infections occurred in critically ill COVID-19 patients requiring intensive care, mechanical ventilation, or extracorporeal support, underscoring the organism's role as an opportunistic pathogen in advanced disease stages.

Su et al. (2020) described a single-patient case report of a 72-year-old man admitted to a tertiary care hospital with quadriplegic Guillain–Barré syndrome and dysautonomia following mild COVID-19. Rapid neurological deterioration necessitated ICU admission and intubation by day 3. Despite the absence of active COVID-19 infection later in the course, the patient developed VAP due to *S. maltophilia*, treated with TMP/SMX, and remained critically ill with profound weakness.

Similarly, Darvishnia et al. (2021) reported a case report of *S. maltophilia* pneumonia complicating severe COVID-19 in a 71-year-old man with Hodgkin's lymphoma. After prolonged intubation and broad-spectrum antimicrobial therapy, *S. maltophilia* pneumonia necessitated tracheostomy and treatment with colistin and TMP/SMX, resulting in partial recovery and ICU discharge after 31 days.

Ishikawa et al. (2022) contributed a single-center case series of four ICU patients with COVID-19-associated *S. maltophilia* infection. All patients received corticosteroids and antiviral therapy, followed by escalation to broad-spectrum antibiotics. *S. maltophilia* was isolated from respiratory specimens in three cases and from both blood and sputum in one. TMP/SMX therapy led to clinical improvement in three patients; one patient died due to COVID-19-associated invasive aspergillosis, highlighting the complexity of co-infections in this setting.

The first documented case of *S. maltophilia* bacteremia in severe COVID-19 was reported by Pek et al. (2021) in a fatal case report. Despite appropriate TMP/SMX therapy, the patient died from refractory septic shock, likely influenced by drug-related adverse effects, renal dysfunction, electrolyte imbalance, and limitations in drug administration during vasopressor-dependent shock.

In contrast, Kuwahara et al. (2022) described a successful case report of catheter-associated *S. maltophilia* bacteremia in a patient with severe COVID-19 supported by extracorporeal membrane oxygenation. Targeted TMP/SMX therapy led to microbiological clearance and recovery, underscoring the importance of early source control and appropriate antimicrobial therapy.

### 4.2 Patients with renal, neurologic, or multisystem complications

Mohamed et al. (2020) presented a case report emphasizing the nephrotoxic risks of TMP/SMX in COVID-19 patients with renal vulnerability. A 64-year-old renal transplant recipient with severe COVID-19 developed *S. maltophilia* pneumonia requiring TMP/SMX, which precipitated acute kidney injury refractory to dialysis and contributed to mortality. This observation aligns with reported acute kidney injury rates of 3–37% in COVID-19 patients (Husain-Syed et al., 2020), reinforcing the need for cautious antimicrobial selection.

### 4.3 Patients with complex microbiological or immunocompromised backgrounds

Öz et al. (2020) reported the first documented case of a tracheostomized patient with COVID-19 successfully managed despite sequential polymicrobial respiratory infections, including *S. maltophilia*. The patient required multiple antibiotic escalations, including meropenem, colistin, and fosfomycin, ultimately achieving recovery after prolonged ICU care. This case highlights the dynamic microbiological evolution in chronically ventilated COVID-19 patients.

Radici et al. (2023) reported a complex case of immuno-oncology involving a patient with refractory mantle cell lymphoma undergoing chimeric antigen receptor T-cell therapy during active COVID-19 infection. The patient developed *S. maltophilia* co-infection alongside COVID-19-associated pulmonary aspergillosis. Prompt antimicrobial and antifungal therapy led to infection resolution and complete lymphoma remission, illustrating favorable outcomes despite extreme immunosuppression.

### 4.4 Non-respiratory and unusual clinical presentations

Beyond pulmonary disease, Bilgic et al. (2021) reported a case series of COVID-19-associated endogenous endophthalmitis, including one case caused by *S. maltophilia*. The affected patient, a 42-year-old man receiving systemic corticosteroids, achieved full visual recovery following intravitreal cefepime, suggesting that immune suppression facilitated ocular dissemination (Zhang et al., 2024).

Alharami et al. (2024) documented a rare pediatric case report of severe *S. maltophilia* stomatitis in a previously healthy 15-year-old girl following COVID-19. Successful treatment with TMP/SMX highlighted that transient immune dysregulation associated with SARS-CoV-2 infection may predispose even immunocompetent individuals to opportunistic infections.

### 4.5 Emerging microbiological mechanisms and indirect pathogenic roles

Soleymani et al. (2024) conducted an observational microbiological study that identified *S. maltophilia* as an endosymbiont of *Acanthamoeba* spp. in COVID-19 patients. This finding suggests a potential indirect pathogenic role, whereby *S. maltophilia* may enhance protozoal virulence, consistent with observations involving other bacterial endosymbionts (Iovieno et al., 2010).

Overall, the case reports reviewed highlight that:

- *S. maltophilia* isolation is clinically significant in patients with COVID-19. In fact, *S. maltophilia* caused monomicrobial infections in most case reports (eight out of 13, 61.5%), and targeted antibiotic therapy resulted in favorable outcomes in most cases (six out of eight, 75%; three “cured,” and three “improved”), while a fatal outcome occurred in two (25%) cases.
- The intubated patients developed VAP in all 10 case reports, confirming that mechanical ventilation is an independent risk factor for *S. maltophilia* infection.
- It is crucial to consider *S. maltophilia* as a potential cause of bacteremia in patients with severe COVID-19 (Pek et al., 2021; Kuwahara et al., 2022). Initiating treatment with TMP-SMX may be preferable when bacteremia is suspected, especially in individuals who are carriers of *S. maltophilia* (Kuwahara et al., 2022). However, significant toxicity risks are possible, particularly in patients with renal dysfunction (Mohamed et al., 2020; Husain-Syed et al., 2020).
- Clinicians should be aware of atypical presentations, such as painful oral ulcers, of *S. maltophilia* opportunistic infections, even in immunocompetent patients, especially during the post-COVID-19 period (Alharami et al., 2024). Notably, COVID-19-associated immune dysregulation may broaden the spectrum of susceptible hosts and clinical manifestations (Radici et al., 2023).
- Polymicrobial and indirect pathogenic mechanisms increasingly complicate management, emphasizing the need for vigilant microbiological surveillance and individualized antimicrobial strategies (Mohamed et al., 2020; Radici et al., 2023; Soleymani et al., 2024).

## 5 Specific risk factors associated with *S. maltophilia* infection in COVID-19 patients

In COVID-19 patients, most available data on risk factors for *S. maltophilia* infection derive from studies focusing on respiratory infections, particularly pneumonia and VAP. Consequently, several risk factors discussed in this section—such as invasive mechanical ventilation, prolonged ICU stay, and the presence of central venous or urinary catheters—should be interpreted primarily as markers of critical illness and ICU-related exposure, rather than as site-specific predictors of non-respiratory infections (e.g., catheter-related bloodstream infections or urinary tract infections), for which robust data in COVID-19 patients remain limited.

The most well-known risk factors for *S. maltophilia* infection include ICU admission, chronic respiratory conditions—predominantly CF—hematologic cancers, mechanical ventilation, chemotherapy-induced neutropenia, organ transplants, HIV infection, hemodialysis, indwelling central venous catheters, recent surgery, trauma, and a history of broad-spectrum antibiotic use (Costa et al., 2025; Koh et al., 2025). However, this pathogen is also increasingly found in critically ill patients, although only a few studies have examined risk factors in ICU patients (Hafiz et al., 2022; Lee et al., 2023).

To our knowledge, there is limited data on the risk factors for *S. maltophilia* pneumonia in critically ill patients with COVID-19, aside from a few isolated case reports. To the best of our knowledge, only two studies have focused solely on *S. maltophilia* (Jacob et al., 2022; Raad et al., 2023). The increase in *S. maltophilia* isolates during the COVID-19 pandemic has been attributed to several factors, including mechanical ventilation, broad-spectrum antibiotics, prolonged hospital stays, and the presence of invasive medical devices.

### 5.1 VAP

Pre-COVID-19 evidence showed that *S. maltophilia* is typically detected in late-onset VAP (Weber et al., 2007). A recent meta-analysis also identified mechanical ventilation as the primary risk factor for *S. maltophilia* pneumonia in non-COVID-19 patients, with prolonged ventilation increasing this risk (Wang et al., 2022).

Patients with severe COVID-19 pneumonia often experience worsening respiratory conditions, which can lead to acute respiratory distress syndrome (ARDS). Therefore, they usually require more extended ventilatory support in the ICU than those with non-COVID-19 ARDS, which explains the high prevalence of *S. maltophilia* in COVID-19 VAP cases (Rouzé et al., 2021). Confirming this, several studies have shown that, although patients with non-COVID-19 ARDS have a high risk (29%−35%) of developing VAP, the occurrence of VAP complications is even higher (36.1%−86%) in patients with severe COVID-19 pneumonia and ARDS, mainly because of the longer duration of ventilator use (Luyt et al., 2020; Rouzé et al., 2021). Additionally, VAP complications can significantly prolong the need for ventilatory support and increase the risk of death in these patients (Nseir et al., 2021).

Although earlier multicenter studies reported an *S. maltophilia* prevalence of nearly 4% among COVID-19 VAP patients (Rouzé et al., 2021; Blonz et al., 2021), recent studies have shown higher prevalence rates. Similarly, a retrospective review of VAP related to severe COVID-19 pneumonia by Morimoto et al. (2024) found that Gram-negative rods accounted for 70% of bacteria isolated from VAP, with *S. maltophilia* and *P. aeruginosa* being the most common (30.4% and 26.1%, respectively). However, comparing *S. maltophilia* with other bacteria revealed no differences in time to VAP onset or ventilator support duration. Accordingly, Shmoury et al. (2023) noted that *S. maltophilia* was significantly more common in VAP than in non-VAP (OR = 3.24, *p* = 0.02). Additionally, the incidence of *S. maltophilia* in VAP in this study (24%) was higher than the 6% reported before the pandemic in a ten-year surveillance study (Kanafani et al., 2019). A single-center study by Raad et al. (2023) reported that endotracheal intubation was associated with a higher incidence of *S. maltophilia* pneumonia (OR = 16.6; 95% CI, 2.14–128.52; *p* = 0.001).

### 5.2 Antibiotic exposure

More than half of ICU patients with SARS-CoV-2 infection were prescribed antibiotics, a rate much higher than the known rate of bacterial co-infections (Abu-Rub et al., 2021), thereby increasing the risk of MDROs emerging and spreading.

In this context, Önal et al. (2023) reported a significant increase in the use of meropenem and ceftriaxone—antibiotics to which *S. maltophilia* is inherently resistant—in all ICUs of a tertiary care university hospital in Turkey after the COVID-19 pandemic. They identified this increase as the primary contributor to the rising rates of *S. maltophilia* bloodstream infections. Morimoto et al. (2024) found that carbapenem use was linked to a higher risk of *S. maltophilia*-induced VAP in COVID-19 patients. Similarly, Huang et al. (2022) and Jacob et al. (2022) observed that most (78.6% and 78.1%, respectively) of COVID-19 patients co-infected with *S. maltophilia* had previously received carbapenem therapy.

In another study, Raad et al. (2023) found that recent exposure to broad-spectrum antibiotics significantly raised the risk of *S. maltophilia* pneumonia in ICU-admitted COVID-19 patients. Notably, the use of meropenem, aminoglycosides, and TMP/SMX was identified as a risk factor for *S. maltophilia* infections. Specifically, COVID-19 patients who developed *S. maltophilia* pneumonia were more frequently treated with combination antibiotic therapy during their ICU stay compared to the control group (89.5% *vs*. 65.2%; *p* = 0.037). They also had higher use of carbapenem (75% *vs*. 46.6%; *p* = 0.02) and aminoglycosides (75% *vs*. 27.2%; *p* < 0.001).

### 5.3 Length of hospital stay

Various factors, including disease prognosis, comorbidities, and resource availability, can complicate the length of hospital stay for COVID-19 patients (Alimohamadi et al., 2022). Specifically, superinfections contributed to more extended hospital stays (Michailides et al., 2023).

Several reports indicated that prolonged hospital stays are a risk factor for *S. maltophilia* infection in COVID-19 patients. Shmoury et al. (2023) reported that *S. maltophilia* was isolated between hospital days 3 and 28, with the highest isolation rates in patients with extended hospital stays (15–28 days). In another study by Raad et al. (2023), the hospital stay for *S. maltophilia* pneumonia in COVID-19 patients was 34 days, compared to 20 days in the control group without COVID-19 (*p* = 0.0001). Similarly, the ICU stay was 33 days, compared to 12 days in the control group (*p* = 0.0001).

Consistent with previous studies, Morimoto et al. (2024) found that patients in the VAP group had a more extended ICU stay (62 days *vs*. 10 days, ICU *vs*. non-ICU; *p* < 0.01), suggesting that prolonged ICU management may increase the risk of VAP caused by *S. maltophilia*. A comparison of *S. maltophilia* with other bacteria showed no difference in ICU length of stay.

### 5.4 Other risk factors

Raad et al. (2023) found that endotracheal intubation (OR = 16.6; 95% CI 2.14–128.52; *p* < 0.001), Foley catheter use (OR = 9.8; 95% CI 1.26–76.1; *p* = 0.009), and the number of central lines placed during ICU stay (*p* = 0.016) significantly increase the risk of *S. maltophilia* infections in COVID-19 patients. In addition to these external risk factors, they identified other important host-related factors that can influence both the likelihood of developing *S. maltophilia* infection and its outcomes. An increased risk was linked to group B and group AB blood types, thrombocytopenia (*p* = 0.034), acute alveolar hemorrhage (*p* < 0.05), and pneumothorax.

## 6 Phenotypic characterization of *S. maltophilia* from COVID-19 patients

Beyond intrinsic antimicrobial resistance, *S. maltophilia* exhibits a complex and multifactorial virulence repertoire that contributes to host persistence and disease severity. A central role is played by diffusible signal factor (DSF)–mediated quorum sensing, which regulates bacterial motility, extracellular enzyme secretion, biofilm formation, and adaptation to environmental stress (Mojica et al., 2022). DSF signaling is controlled by the *rpf* gene cluster, whose allelic variants (*rpf-1* and *rpf-2*) exhibit distinct phenotypic profiles, including differences in virulence expression and biofilm strength. In particular, *rpf-1* variants display more efficient DSF signaling and enhanced biofilm formation, whereas *rpf-2* variants show attenuated DSF production and altered regulatory responses (Mojica et al., 2022).

Another relevant virulence-related feature is the production of outer membrane vesicles (OMVs), which act as carriers of enzymes, signaling molecules, and other factors involved in host–pathogen interactions. OMV release has been shown to increase in response to antibiotic exposure—such as imipenem and ciprofloxacin—as well as to DSF signaling, suggesting a stress-induced adaptive mechanism that may promote persistence under antimicrobial pressure (Mojica et al., 2022). In addition, recent genomic and phylogenetic studies have identified lineage-specific virulence determinants, including the transcriptional regulator SmoR and the catalase KatA, which are unevenly distributed among major *S. maltophilia* lineages (e.g., Sm6, Sm4, Sm3) and may underlie strain-dependent differences in pathogenic potential concerning quorum sensing, swarming motility, and persistence to disinfectants (Mojica et al., 2022).

Collectively, these mechanisms converge on the organism's capacity to establish persistent biofilms on abiotic surfaces, such as ventilator circuits and other medical devices. This trait is particularly relevant in ICU and VAP settings, where prolonged device exposure and antibiotic selective pressure favor long-term colonization, polymicrobial communities, and difficult-to-eradicate infections. Integrating these virulence mechanisms provides essential biological context for understanding the epidemiological and clinical behavior of *S. maltophilia* in critically ill COVID-19 patients.

To the best of our knowledge, only a few studies have focused on the phenotypic characterization of *S. maltophilia* strains isolated from COVID-19 patients, primarily regarding variations in antibiotic resistance and, to a lesser extent, biofilm-forming ability.

### 6.1 Antibiotic resistance

The overuse of antibiotics during the COVID-19 pandemic disrupted antimicrobial stewardship programs and worsened global antimicrobial resistance (Langford et al., 2023). *S. maltophilia* is considered an MDRO primarily because of its extensive intrinsic resistance, mediated by chromosomally encoded determinants such as the L1 and L2 β-lactamases and multiple efflux pump systems (e.g., SmeDEF), rather than by the widespread acquisition of resistance genes. However, additional acquired resistance may occur under antibiotic selective pressure (Mojica et al., 2022).

Increases in MDROs have been reported in multiple studies during the COVID-19 pandemic, involving pathogens associated with HAIs, such as carbapenemase-resistant *K. pneumoniae, P. aeruginosa, A. baumannii*, and MRSA (Son et al., 2021; Lai et al., 2021). Notably, Son et al. (2021) identified *S. maltophilia* as the most common MDRO isolated in a cohort of 152 COVID-19 pneumonia patients across 10 hospitals in the Republic of Korea between February and May 2020. Use of systemic corticosteroids after COVID-19 diagnosis (aOR: 15.07; 95% CI: 2.34–97.01; *p* = 0.004) and long-term care facility stay before diagnosis (aOR: 6.09; 95% CI: 1.02–36.49; *p* = 0.048) were linked to MDRO isolation. Similarly, Fu et al. (2020) reported five cases of critically ill COVID-19 patients with secondary infections, where ESBL-producing *K. pneumoniae, S. maltophilia, B. cenopacia*, and *P. aeruginosa* were identified as the causative pathogens.

A concern is the rising resistance to TMP/SMX and levofloxacin, which are considered the primary treatments for *S. maltophilia* infections (Monardo et al., 2025). In COVID-19 patients, TMP/SMX is typically the first-line treatment for mild *S. maltophilia* infections, with levofloxacin as an alternative if TMP/SMX resistance develops, hypersensitivity occurs, or acute kidney injury develops (Ishikawa et al., 2022). de Souza et al. (2025) reported resistance rates of 22.2% for TMP/SMX and 11.1% for levofloxacin among nine *S. maltophilia* strains isolated, from June 2021 to February 2022, from patients with confirmed COVID-19 and without COVID-19 coinfection at an army hospital in Rio de Janeiro. Higher resistance to TMP/SMX was noted by Burastero et al. (2022), with half of the isolates causing VAP resistant to TMP/SMX.

Raad et al. (2023) evaluated the antibiotic susceptibility of 40 *S. maltophilia* isolates from COVID-19 patients. They found that isolates from initial pulmonary infections showed 35% resistance to levofloxacin, which increased to 53.8% during the second infection. Patients with *S. maltophilia* superinfection were more likely to have been exposed to fluoroquinolones compared to the control group, which may have contributed to the development of acquired resistance to these antibiotics. In contrast, they observed high sensitivity to TMP/SMX in the first nosocomial pneumonia (95%), which remained at 92.3% in subsequent infections.

Hafiz et al. (2022) observed a higher resistance rate to ceftazidime (62.1%), followed by levofloxacin (14.8%), and then TMP/SMX (4.1%) among 393 *S. maltophilia* isolates collected from January 2019 to December 2021 at King Fahad Medical City in Riyadh, Saudi Arabia. Analyzing antimicrobial resistance in secondary bacterial infections among patients hospitalized with COVID-19 in Wuhan, Li et al. (2020) reported resistance rates of 90% and 30% for ceftazidime and levofloxacin, respectively. In contrast, no resistance to TMP/SMX was observed.

Contrary to other studies, Cornejo-Juárez et al. (2023) found no significant changes in antibiotic resistance in *S. maltophilia* strains isolated during the pre-pandemic period (2018 and 2019) and the pandemic period (April–December 2020 and 2021) at an oncology hospital in Mexico City.

Overall, these findings underscore the urgent need for research and surveillance to assess antimicrobial resistance in *S. maltophilia* among COVID-19 patients, at both the individual and population levels, to inform effective clinical management. Notably, there was little to no resistance reported for minocycline, cefiderocol, and colistin (Li et al., 2020; Raad et al., 2023; de Souza et al., 2025), making them promising options for future therapy—especially when an antibiotic switch is necessary due to treatment failure or complications with TMP/SMX, as seen in ICU settings (Pek et al., 2021).

### 6.2 Biofilm formation

The production of biofilm is a crucial aspect of *S. maltophilia* virulence, as it enhances resistance to antibiotics and antiseptic solutions, challenges the host's immune defenses, and contributes to the progression of CF lung disease and other chronic respiratory conditions (Brooke, 2021; Pompilio et al., 2020). Supporting this, biofilms are estimated to be involved in 65% of HAIs caused by *S. maltophilia* (Brooke, 2021).

de Souza et al. (2025) evaluated the ability of *S. maltophilia* strains, isolated from various environmental sources at an army hospital in Rio de Janeiro, to form biofilms. Interestingly, most strains could adhere and form significant biofilms, even at environmental temperatures, on stainless steel and polystyrene surfaces. Additionally, exposure to 0.5% peracetic acid, 70% alcohol, and 5th-generation/stabilized polymeric biguanide quaternary ammonium did not significantly reduce biofilm formation.

Attempting to understand specific features of biofilms formed by *S. maltophilia* from COVID-19 patients, Strateva et al. (2023) investigated how well biofilms form in strains isolated from COVID-19 patients compared to those from non-COVID-19 patients. Although biofilm levels were similar between the two groups, the non-COVID group showed a wider range of biofilm amounts, likely because fewer specimen types were available from COVID-19 patients, with *S. maltophilia* recovered mainly from respiratory samples.

Overall, these findings suggest that the increased isolation of *S. maltophilia* in COVID-19 patients may be attributed to the bacterium's ability to colonize and form biofilms on medical devices, particularly those used in endotracheal intubation and mechanical ventilation, procedures commonly employed for these patients (Cronin et al., 2022).

This evidence highlights the importance of investigating potential sources of infection, including hospital water supplies, faucets, ice machines, oxygen humidification reservoirs, and any hospital products shared among patients (e.g., dialyzer effluent, disinfectant solutions). Reviewing disinfection protocols is also crucial for identifying violations, such as the use of non-sterile water during these procedures.

## 7 Clinical significance of *S. maltophilia* in COVID-19 patients

*S. maltophilia* typically causes nosocomial infections, especially in immunocompromised populations, although community-acquired infections have also been reported. Respiratory infections, such as pneumonia, and exacerbations of chronic obstructive pulmonary disease are the most common (Brooke, 2021). It also frequently occurs in patients with CF, tracheostomized patients, or those on mechanical ventilation. Besides pulmonary infections, other common infections, including bloodstream infections, meningitis, soft tissue and skin infections, catheter-associated infections, and urinary tract infections, have also been reported (Brooke, 2021).

A major challenge in interpreting the available data is distinguishing respiratory colonization from true infection, as several studies classified *S. maltophilia* infection solely on culture positivity, often in the absence of standardized clinical, radiological, or quantitative microbiological criteria. This issue is particularly relevant in mechanically ventilated COVID-19 patients, in whom prolonged ICU stay and device exposure favor airway colonization.

The pathogenesis of *S. maltophilia* infections is complex due to its numerous potent and potential virulence factors. Among these, the natural and acquired resistance to many antibiotics used in healthcare, along with its ability to colonize—by growing as antibiotic-resistant biofilms—medical devices such as those used for mechanical ventilation, are the most significant (Pompilio et al., 2020; Demirbuga et al., 2024).

Despite the increased detection of *S. maltophilia* during the pandemic, its exact role in causing disease in COVID-19 patients remains unclear. *S. maltophilia* is known to colonize the respiratory tract of individuals with chronic respiratory conditions. Therefore, it is difficult to determine whether *S. maltophilia* is the cause of pneumonia or merely a colonizer, especially in polymicrobial cases where *S. maltophilia* is often present (Jacob et al., 2022). Additionally, most isolates originate from ventilated patients, and diagnosing VAP is notoriously difficult, with both clinical and bacteriological methods having limited sensitivity and specificity (American Thoracic Society Infectious Diseases Society of America, 2005).

Several pieces of evidence demonstrate that isolation is clinically significant in patients with COVID-19. First, most patients showed signs of new organ dysfunction—such as hypotension, worsening breathing, and fever. Second, *S. maltophilia* is associated with high morbidity and mortality, especially when causing bacteremia (Pek et al., 2021; Burastero et al., 2022; Kuwahara et al., 2022; Choi et al., 2023; Raad et al., 2023; Satjawattanavimol et al., 2023). Notably, Raad et al. (2023) not only showed that in COVID-19 patients *S. maltophilia* infections were associated with a rise in median death rate (from 40% to 60%), but also that this specific superinfection increased the chances of long-term complication rate at discharge, such as tracheostomy (28.6% *vs*. 4.8%) and oxygen dependence (57% *vs*. 42%). Third, administering appropriate antibiotics targeting *S. maltophilia* often improved the patient's condition and reduced mortality, even after accounting for other factors that predict mortality (Darvishnia et al., 2021; Jacob et al., 2022; Kuwahara et al., 2022; Morimoto et al., 2024; Zhang et al., 2024; Alharami et al., 2024).

On the other hand, limited evidence suggests that *S. maltophilia* might be a bystander colonizer or, rather, a marker of disease in COVID-19 patients with more severe lung issues caused by other pathogens. In this context, some studies reported that the clinical decline leading to death in certain patients was associated with co-occurring bacterial infections, such as *Clostridium perfringens* bacteremia, ESBL-producing *K. pneumoniae*, MRSA, and MDR *A. baumannii* (Nasir et al., 2020), or invasive aspergillosis (Ishikawa et al., 2022). Furthermore, Morimoto et al. (2024) observed that, although antibiotic therapy improved clinical and imaging outcomes and reduced sputum *S. maltophilia* load in most patients, in one case, the clinical condition and imaging findings improved even without antibiotics.

Another potential scenario, already observed in CF patients (Pompilio et al., 2015), involves *S. maltophilia* acting as an active part of polymicrobial bacterial pulmonary communities. It may provide a specific “fitness advantage” to nearby key pathogens, such as *P. aeruginosa*, under conditions of chronic infection, increasing their virulence and leading to pulmonary exacerbations. Supporting this, Yin et al. (2017) found that co-infection with *S. maltophilia* can have a synergistic effect on mortality, as observed in critically ill patients with *P. aeruginosa*.

## 8 Antibiotic therapy of *S. maltophilia* infections in COVID-19 patients

Although the significance of *S. maltophilia* in critically ill patients has not been thoroughly examined, evidence from the literature strongly indicates that COVID-19 patients, particularly those admitted to the ICU, are becoming an emerging niche for *S. maltophilia* infections.

No specific quantitative cut-offs have been validated to determine the clinical relevance of *S. maltophilia* in the respiratory tract, so all potential pathogens must be assessed. Despite this, in patients undergoing prolonged mechanical ventilation, this may lead to the selection of *S. maltophilia* and necessitate targeted treatment.

Treating *S. maltophilia* is challenging because this pathogen exhibits high levels of inherent or acquired resistance to many antibiotics, which limits treatment options, and because the underlying illness renders patients more susceptible to these infections. A recent comprehensive narrative review on this topic was published by Monardo et al. (2025).

The Infectious Diseases Society of America (IDSA) guidelines do not specify a recommended antibiotic regimen for *S. maltophilia* infections in patients with COVID-19 due to limited evidence on optimal treatment. The general approach suggested by IDSA for the treatment of infections caused by *S. maltophilia* consists of either of 2 options (Tamma et al., 2024). The first is combination therapy with at least 2 active agents (i.e., cefiderocol, minocycline, TMP-SMX, or levofloxacin)—listed in order of preference. The nephrotoxic potential of TMP/SMX must be considered in patients with prior renal impairments, as it can cause acute kidney injury requiring hemodialysis and may be fatal (Ishikawa et al., 2022; Mohamed et al., 2020). Alternatively, the combination of ceftazidime-avibactam (CZA) and aztreonam can be administered (Tamma et al., 2024). Using CZA without aztreonam is not recommended, as most isolates are resistant to this combination due to metallo-beta-lactamase L1 and serine-beta-lactamases L2 (Tamma et al., 2024).

Few studies, mostly case series, have reported the effectiveness of antibiotic therapy for treating *S. maltophilia* infections in COVID-19 patients. In the case series discussed above (see “Case reports” section), *S. maltophilia* monomicrobial infections—mainly VAPs, except for one case of endophthalmitis—were treated with targeted antibiotic therapy, most often using TMP/SMX (Su et al., 2020; Mohamed et al., 2020; Pek et al., 2021; Ishikawa et al., 2022; Kuwahara et al., 2022; Alharami et al., 2024), cefepime (Bilgic et al., 2021), or colistin combined with TMP/SMX (Darvishnia et al., 2021). This led to a favorable outcome—either cure or improvement in clinical status—in most cases (six out of eight, 75%; three were cured, and three showed improvement). The death was recorded in two (25%) cases (Mohamed et al., 2020; Pek et al., 2021), when TMP/SMX alone was used. However, this unfavorable outcome was attributed to acute kidney injury caused by the renal toxicity of TMP/SMX, rather than its inefficacy.

When *S. maltophilia* was isolated alongside other pathogens, antibiotic therapy consistently led to favorable outcomes: colistin and fosfomycin with *P. aeruginosa* (Öz et al., 2020), TMP/SMX and voriconazole with COVID-19-associated aspergillosis pneumonia (CAPA) (Ishikawa et al., 2022), TMP/SMX and meropenem with *Klebsiella aerogenes* and *Enterococcus faecalis* (Ishikawa et al., 2022), TMP/SMX, levofloxacin, and minocycline with CAPA (Ishikawa et al., 2022), and moxifloxacin and isavuconazole with *K. pneumoniae* and CAPA (Radici et al., 2023).

In seven COVID-19 patients with VAP and suspected *S. maltophilia* infection, Morimoto et al. (2024) reported prescribing TMP/SMX, levofloxacin, minocycline, or ciprofloxacin, alone or in combination, based on susceptibility testing. The therapy led to improved clinical and imaging findings, along with a decrease in *S. maltophilia* load in sputum.

In another study, Burastero et al. (2022) documented six cases of COVID-19-associated VAP caused by *S. maltophilia*, five of which were polymicrobial, with 50% exhibiting resistance to TMP/SMX. TMP/SMX was administered to three patients, while two received a combination of TMP/SMX and fosfomycin. In one case, CZA was given as monotherapy. The only patient who died after 7 days of treatment was treated with CZA alone.

Overall, these findings indicate that TMP/SMX remains the preferred antimicrobial treatment for COVID-19 patients with *S. maltophilia* infections, even when multiple pathogens are involved. However, the recent rise in resistance rates to this drug and its nephrotoxic potential requires careful use and ongoing monitoring.

A potential way to improve antibiotic use in clinical practice and reduce bacterial resistance is to use rapid microbiological diagnostics to identify pathogens and test antimicrobial susceptibility. For instance, using a molecular method such as real-time PCR to detect multiple respiratory pathogens can reduce the time required for pathogen identification, thereby decreasing the likelihood of false-negative cultures (Yang et al., 2021; Foschi et al., 2021). This method can expedite the initiation of antimicrobial therapy, ultimately improving outcomes in critically ill COVID-19 patients. Additionally, multiplex testing can identify key markers of antibiotic resistance, supporting antimicrobial stewardship.

## 9 Criticisms and limitations of the reviewed studies

Currently, many microorganisms are often reported as co-pathogens in critically ill patients with COVID-19. Based on existing reports, about 50% of COVID-19 deaths have been linked to secondary bacterial infections (Zhou et al., 2020). However, data on the microbiological ecology in these patients, especially regarding the prevalence and clinical importance of *S. maltophilia*, are limited and sometimes inconsistent. Differences in findings across the studies reviewed here may result from several factors discussed in this section.

- A significant temporal limitation of the available literature is that most studies were conducted during the pre-Omicron phases of the COVID-19 pandemic (corresponding mainly to the Alpha and Delta waves), when ICU management frequently involved prolonged invasive ventilation, high extracorporeal membrane oxygenation utilization, and widespread use of corticosteroids and IL-6 inhibitors. These clinical conditions differ substantially from those observed during the Omicron era, potentially influencing the epidemiology of opportunistic pathogens such as *S. maltophilia*. Therefore, the findings of this review should be interpreted in light of evolving viral variants and changing clinical practices, warranting future studies to assess whether the incidence and clinical impact of *S. maltophilia* infections have changed in Omicron-era ICUs.
- Nearly all the studies reviewed were retrospective (26 out of 27, 96.3%) and conducted at a single center (19 out of 27, 70.4%). Potential bias could not be avoided in single-center retrospective studies, which may lead to outcomes that do not accurately reflect those from other institutions or regions. Additionally, retrospective studies could not control for confounding factors or variables that might affect the clinical course and disease outcomes, such as missing data on patient comorbidities, prior bacterial colonization, antibiotic use before pneumonia onset, and mechanical ventilation duration in patients who developed VAP. Finally, because these studies were retrospective, epidemiological data rather than patients' clinical details were primarily evaluated.
- Enrolling only hospitalized patients with COVID-19 could have led to a biased sample because of the high severity of the disease.
- The sample size was often determined incidentally and was not designed to be statistically sufficient to detect the effects of complications from bacterial infections on severe illness and death. Furthermore, the high variability in the number of study subjects may help explain the disagreement. In this review, the number of patients enrolled in the studies ranged from 20 (Yang et al., 2021) to 1,863 (Nakagawara et al., 2023) (Figure 3).
- Different diagnostic criteria for hospital-acquired superinfections may cause variations in findings. For example, several studies define them as those “diagnosed at least 48 hours after hospital admission” (Sang et al., 2021; Signorini et al., 2021; Huang et al., 2022; Nakagawara et al., 2023), while Li et al. (2020) described them as cases where “patients showed clinical characteristics of bacterial infections, and at least one positive bacterial etiology was found from qualified microbiological specimens (such as sputum, endotracheal aspirate, bronchoalveolar lavage fluid, blood, or urine) after SARS-CoV-2 infection” without considering the timing of diagnosis.
- Several studies defined *S. maltophilia* infection solely based on respiratory culture positivity, frequently without standardized clinical, radiological, or quantitative microbiological criteria. This limitation complicates the distinction between true infection and respiratory colonization, particularly in mechanically ventilated COVID-19 patients, in whom prolonged ICU stays, exposure to invasive devices, and polymicrobial airway colonization are common.
- There was no standardized protocol for screening COVID-19 patients for different types of HAI, and most diagnosed cases relied on the clinical judgment of the treating physicians to order microbiological tests. The lack of validated criteria can make it challenging to distinguish between nosocomial pneumonia, especially VAP, and the progression of COVID-19.
- The diagnosis of VAP is not always confirmed by an independent committee, which often leads to overestimating its occurrence. Additionally, although COVID-19 patients tested positive by reverse transcription PCR, it is not definitive that all had pneumonia caused by SARS-CoV-2, since some may have been on invasive mechanical ventilation for other reasons, such as cardiogenic shock or severe pancreatitis.
- The prevalence of *S. maltophilia* may also have been influenced by the different bacterial identification techniques used across various studies. Culture analysis was the most common method among the reviewed studies (24 out of 27; 88.9%). In contrast, culture-independent techniques, such as multiple real-time PCR, multiplex PCR, and real-time reverse transcription loop-mediated isothermal amplification, although they offer higher sensitivity in detecting potential pathogens, were employed in only three studies (Li et al., 2020; Yang et al., 2021; Foschi et al., 2021). The increased sensitivity of molecular methods enables rapid, accurate detection of more bacteria in a sample, particularly at low bacterial loads (Li et al., 2020; Foschi et al., 2021). However, this can also complicate distinguishing between colonization and true infection, as seen with *S. maltophilia*.
- Management practices may vary across different facilities. Specifically, antibiotic use and treatment methods are not standardized, which could have affected clinical results.
- Antimicrobial susceptibility tests (AST) results are also frequently missing or incomplete, particularly in overwhelmed clinical settings, underscoring the need for greater emphasis on longitudinal monitoring of MDR patterns and their temporal evolution, especially among patients with COVID-19. This scenario is further complicated by several limitations that restrict confidence in reported AST results, as summarized by Mojica et al. (2022). Breakpoints differ among CLSI, EUCAST, and the U.S. Food and Drug Administration, leading to inconsistent susceptibility classification. Commercial AST systems show high rates of very major and major errors, raising concern for false-susceptible or false-resistant results. Many breakpoints were derived from small isolate datasets, and reference broth microdilution methods have largely been extrapolated from *P. aeruginosa*, despite key biological differences. In addition, robust pharmacokinetic/pharmacodynamic data are lacking for most agents, and clinical failures have been reported even when *in vitro* results predict susceptibility.
- The rate of bacterial coinfection may vary depending on how many times the specimens are collected. In this regard, Yang et al. (2021) demonstrated that multiple attempts at specimen collection are associated with a higher prevalence of bacterial co-infections.
- A prolonged collection period prevents further analysis of the data, which involves classification by isolate type and patient subgroup.

Future studies should address these limitations to gain the essential knowledge needed to guide empirical antimicrobial therapy in this population and inform policies related to antimicrobial stewardship, infection prevention, and control.

## 10 Conclusions

The findings from the studies reviewed here have consistently demonstrated that *S. maltophilia* acts as an opportunistic pathogen in individuals with multiple chronic conditions and weakened immune systems. Higher rates of *S. maltophilia* detection have indeed been observed during the COVID-19 pandemic, emphasizing its growing significance.

The higher prevalence is likely due to selective pressure from the overuse of broad-spectrum antibiotics, such as cephalosporins and carbapenems, as well as reserve antibiotics like colistin, especially in critical care settings. Additionally, the COVID-19 pandemic has increased demand for invasive medical equipment, such as ventilators, and has led to more extended hospital stays, particularly in ICUs. These conditions, recognized as risk factors for *S. maltophilia* infection, likely contributed to the rise in *S. maltophilia* cases due to excessive antibiotic use.

*S. maltophilia* coinfections and secondary infections in COVID-19 patients are closely associated with disease severity and high mortality rates. Therefore, it is crucial to identify patients at high risk of *S. maltophilia* nosocomial infection to manage COVID-19 cases effectively, enabling early detection and prompt treatment. This is particularly important for a multidrug-resistant species like *S. maltophilia*, highlighting the need to enhance policies and practices to prevent the development of antimicrobial resistance. Although TMP/SMX and levofloxacin remain the gold standard treatments for *S. maltophilia* infections in COVID-19 patients, rising resistance rates underscore the importance of monitoring antimicrobial resistance patterns in these patients.

Large-scale, multicenter, prospective cohort studies involving a larger number of COVID-19 patients from hospitals across different regions, using standardized clinical and diagnostic protocols, are needed to better understand the relationship between *S. maltophilia* and SARS-CoV-2, as well as its impact on patient outcomes. The results of these studies could be crucial for informing empirical treatment choices, developing effective COVID-19 management strategies, and implementing targeted measures to prevent or reduce superimposed infections in the future.
